# Expression of *Ccl11* Associates with Immune Response Modulation and Protection against Neuroinflammation in Rats

**DOI:** 10.1371/journal.pone.0039794

**Published:** 2012-07-16

**Authors:** Milena Z. Adzemovic, Johan Öckinger, Manuel Zeitelhofer, Sonja Hochmeister, Amennai Daniel Beyeen, Atul Paulson, Alan Gillett, Melanie Thessen Hedreul, Ruxandra Covacu, Hans Lassmann, Tomas Olsson, Maja Jagodic

**Affiliations:** 1 Neuroimmunology Unit, Department of Clinical Neuroscience, Center of Molecular Medicine, Karolinska Institutet, Stockholm, Sweden; 2 Department of Neuroimmunology, Center for Brain Research, Medical University of Vienna, Vienna, Austria; 3 Department of Neurology, Medical University of Graz, Graz, Austria; Universidad Federal de Santa Catarina, Brazil

## Abstract

Multiple sclerosis (MS) is a polygenic disease characterized by inflammation and demyelination in the central nervous system (CNS), which can be modeled in experimental autoimmune encephalomyelitis (EAE). The *Eae18b* locus on rat chromosome 10 has previously been linked to regulation of beta-chemokine expression and severity of EAE. Moreover, the homologous chemokine cluster in humans showed evidence of association with susceptibility to MS. We here established a congenic rat strain with *Eae18b* locus containing a chemokine cluster (*Ccl2, Ccl7, Ccl11, Ccl12* and *Ccl1*) from the EAE- resistant PVG rat strain on the susceptible DA background and utilized myelin oligodendrocyte glycoprotein (MOG)-induced EAE to characterize the mechanisms underlying the genetic regulation. Congenic rats developed a milder disease compared to the susceptible DA strain, and this was reflected in decreased demyelination and in reduced recruitment of inflammatory cells to the brain. The congenic strain also showed significantly increased *Ccl11* mRNA expression in draining lymph nodes and spinal cord after EAE induction. In the lymph nodes, macrophages were the main producers of CCL11, whereas macrophages and lymphocytes expressed the main CCL11 receptor, namely CCR3. Accordingly, the congenic strain also showed significantly increased *Ccr3* mRNA expression in lymph nodes. In the CNS, the main producers of CCL11 were neurons, whereas CCR3 was detected on neurons and CSF producing ependymal cells. This corresponded to increased levels of CCL11 protein in the cerebrospinal fluid of the congenic rats. Increased intrathecal production of CCL11 in congenic rats was accompanied by a tighter blood brain barrier, reflected by more occludin^+^ blood vessels. In addition, the congenic strain showed a reduced antigen specific response and a predominant anti-inflammatory Th2 phenotype. These results indicate novel mechanisms in the genetic regulation of neuroinflammation.

## Introduction

Multiple sclerosis (MS) is an inflammatory demyelinating disease of the central nervous system (CNS), causing loss of sensory and motor functions in affected individuals. The disease is polygenic, and several genes contributing to susceptibility have been identified and confirmed [1], [2]. Characterization of the molecular mechanisms that mediate influence of MS risk genes can provide novel insights into disease pathogenesis. For example, genetically regulated expression of CD58, a cell adhesion molecule expressed primarily on macrophages, is protective due to enhanced activation of regulatory T cells [3]. However, the genes identified so far still explain only a part of the disease heritability [4]. In addition, the underlying mechanisms are described only for a limited set of genes. To further dissect the genetic and pathological mechanisms of neuroinflammation, several animal models are used. Myelin oligodendrocyte glycoprotein (MOG)-induced experimental autoimmune encephalomyelitis (EAE) is a well characterized animal model of MS, sharing several important features including T helper 1 (Th1), Th17 and B-cell involvement as well as histopathological characteristics [5], [6], [7], [8].

Previous linkage analyses in experimental crosses and congenic strains have identified a quantitative trait locus (QTL) on rat chromosome 10 [9], [10] and the homologous region on mouse chromosome 11 [11] as a candidate region for regulation of neuroinflammation. Initial linkage scans identified regions too large to allow for candidate gene investigation, but we have been able to restrict the QTL to a 0.88 Mb interval known as *Eae18b*, which includes a cluster of chemokine genes (*Ccl2, Ccl7, Ccl11, Ccl12* and *Ccl1*) using high-resolution mapping approaches [12], [13]. Chemokines are small, secreted proteins involved in chemotaxis and activation of a variety of immune cells, acting through their corresponding receptors (reviewed in [14]). We have previously demonstrated that expression of these chemokine genes during EAE is regulated by the genotype in the region, i.e. *cis*-regulation [13].

In this study we sought to confirm the impact of the *Eae18b* locus on neuroinflammation and identify the chemokine(s) in the gene cluster that underlies the genetic regulation of EAE. Through selective backcrossing, we created a congenic strain (DA.PVG-*Eae18b*) that comprises alleles from the EAE-resistant Piebald Virol Glaxo (PVG) strain on the EAE-susceptible Dark Agouti (DA) background. These two strains differ in chemokine gene expression, but have the identical exonic sequences in the chemokine genes. We studied the impact of *Eae18b* locus on EAE development and observed a milder disease and a higher expression of CCL11 mRNA and protein levels in inguinal lymph nodes in the *Eae18b* congenic strain. The upregulation of CCL11 and its main receptor CCR3 was associated with a Th2-like immune response, which could skew the inflammatory response towards an anti-inflammatory state. In addition, we found increased levels of CCL11 in the cerebrospinal fluid (CSF) of the congenic strain, which possibly influences the blood brain barrier (BBB) integrity.

## Results

### *Eae18b* Congenic Animals Display Milder Disease

**Figure 1:**
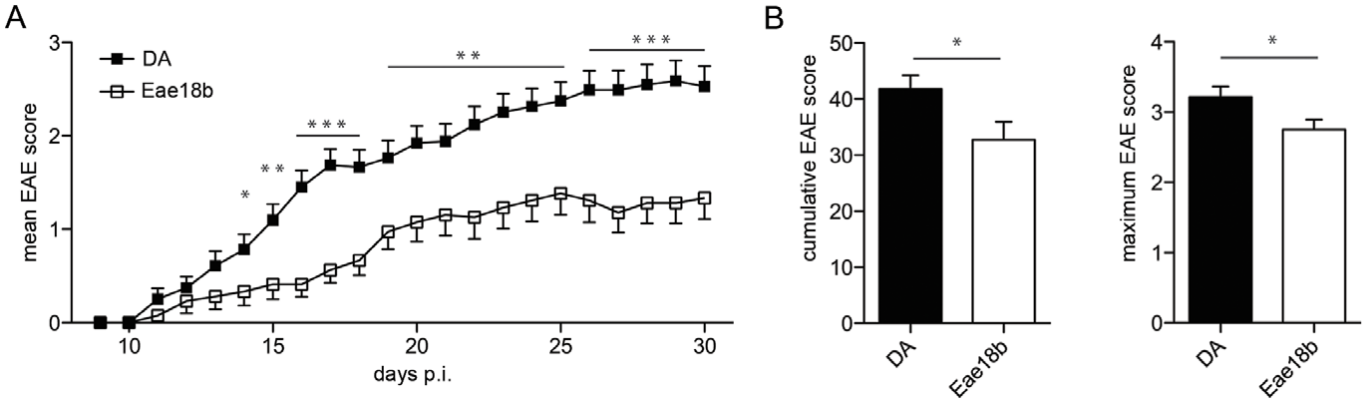
Congenic DA.PVG-*Eae18b* rats display clinically milder EAE. (A) Mean daily EAE score from 51 DA and 39 DA.PVG-*Eae18b* (*Eae18b*) rats, immunized with MOG, p.i: post immunization. The graph includes daily mean scores from all immunized animals in two independent experiments. (B) Mean cumulative EAE score and mean EAE maximum score in affected rats (44 DA and 20 DA.PVG-*Eae18b* animals). Error bars represent SEM, and (*) indicates p-value <0.05, (**) indicates p-value <0.01, (***) indicates p-value <0.001.

To confirm the biological effect of *Eae18b* in EAE, we immunized congenic DA.PVG-*Eae18b* and control DA rats with MOG. The congenic strain developed an overall milder disease with lower mean EAE score from day 14 post immunization (p.i.) until the end of the experiment compared to the DA strain (Figure 1A). The congenic strain showed a reduced incidence of disease with an overall incidence of 51% compared to 86% in the DA strain (p-value: 0.0004, Fischer exact test). In addition, the affected animals in the congenic strain also displayed a reduced severity of disease, reflected in significantly lower cumulative and maximum EAE scores (Figure 1B).

### *Ccl11* is Highly Expressed in *Eae18b* Congenic Animals during EAE

**Figure 2:**
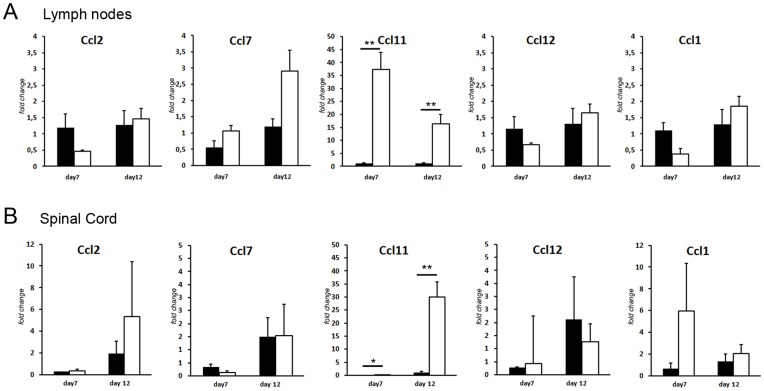
*Eae18b* congenic animals significantly upregulate *Ccl11* mRNA. qPCR was used to measure mRNA expression of *Ccl2, Ccl7, Ccl11, Ccl12* and *Ccl1* in draining lymph nodes (A) and spinal cord tissue (B) on day 7 and 12 p.i. *Ccl11* mRNA was significantly upregulated in the congenic strain in both lymph node and spinal cord tissue at both time-points. Five to eight animals per strain were used at each time-point and experiments were repeated three times. Error bars represent SEM, statistics were calculated using Mann-Whitney non-parametric test, (*) indicates p-value <0.05, (**) indicates p-value <0.01.

We have previously demonstrated a *cis*-regulation of chemokine transcription during EAE [13]. Therefore we sought to investigate the role of the chemokine expression in the *Eae18b* region in the regulation of inflammation. In order to study the kinetics of the expression of the chemokine genes during the induction of disease, mRNA levels of *Ccl2, Ccl7, Ccl11, Ccl12* and *Ccl1* were measured in lymph nodes and spinal cords from parental DA and PVG.av1 rats, at day 0, 5, 7 and 12 days after MOG immunization, using real-time quantitative PCR (qPCR). The expression analysis in the parental strains (DA and PVG.av1) revealed different regulation of *Ccl11* and *Ccl12* on day 7 and 12 p.i. in the lymph nodes and on day 12 p.i. in the spinal cord (Figure S1A and B). Having established the time points when the parental strains differ in expression, we further analyzed the mRNA expression of the chemokine genes in the DA.PVG-*Eae18b* congenic strain compared to littermate DA controls. In lymph nodes and spinal cord, *Ccl11* was significantly upregulated in the congenic animals compared to the DA strain on both day 7 and 12 p.i. (Figure 2A and B). The other chemokine genes included in the cluster did not show any significant differential expression. These findings directly associate the regulation of *Ccl11* expression to the genetic region defined by the congenic fragment.

### *Ccl11* is Predominantly Expressed by Macrophages in the Lymph Nodes

**Figure 3:**
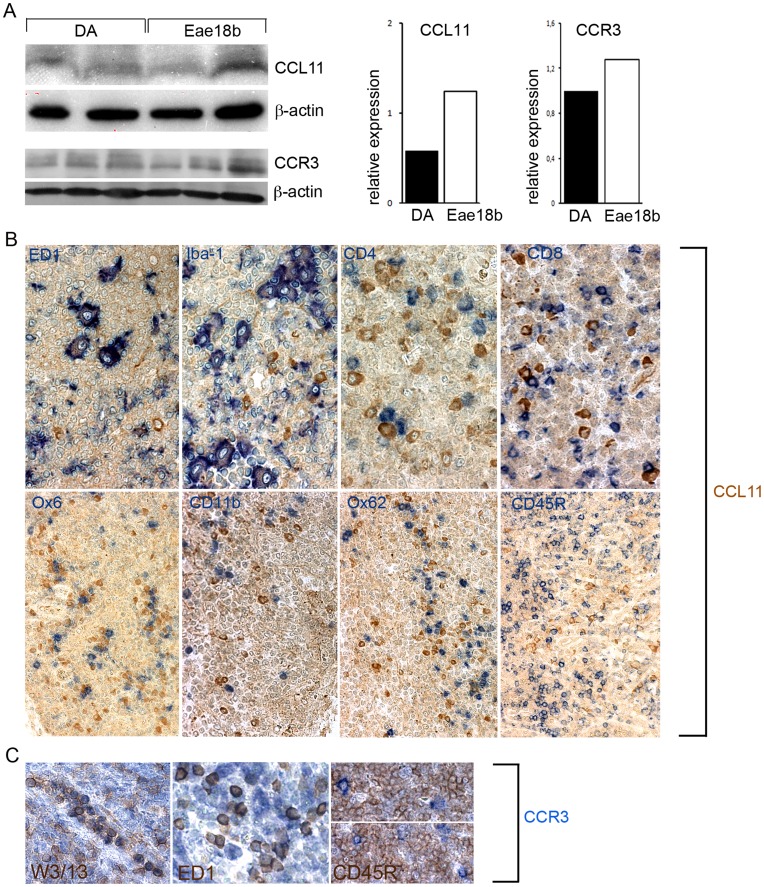
CCL11 and CCR3 protein levels in the inguinal lymph nodes. (A) Western blot analysis of CCL11 and its main receptor CCR3 in inguinal lymph nodes on day 7 p.i. Protein levels of both targets were increased in the congenic animals compared to the controls (data normalized to β- actin). CCL11, CCR3 and β- actin shown in original representative blots and graphically; three blots performed for each target within two separate EAE experiments. (B) Co-staining with antibodies directed against CCL11 and other immune cell types on paraffin cross sections of inguinal lymph nodes performed on day 7 p.i. in the congenic *Eae18b* strain. CCL11 co-localized with the macrophage/microglia markers ED1 and Iba-1 (first two images from the left, upper row). CCL11 was not expressed by CD4^+^ and CD8^+^ T cells (last two images, upper row). CCL11 was also not detected in MHC class II^+^ (Ox6^+^) and CD11b^+^ populations, neither in Ox62^+^ dendritic, nor in CD45RA^+^ B cells (second row, from left to right). (C) The main CCL11 receptor CCR3 was detected on W3/13^+^ T lymphocytes and ED1^+^ macrophages, but not on CD45RA^+^ B cells (five congenic and five control rats analyzed, two whole inguinal lymph nodes per each animal).

In order to confirm that the increased *Ccl11* and *Ccr3* mRNA expression also resulted in elevated CCL11 and CCR3 protein levels, respectively, we performed Western Blot and extensive immunohistochemical (IHC) analysis on cross-sections from draining inguinal lymph nodes harvested on day 7 p.i. Western blot analysis revealed that the lymph nodes of the congenic strain expressed increased levels of CCL11 and CCR3 protein (Figure 3A). Thereafter, we aimed to elucidate the cellular source of the increased CCL11 and CCR3 production in the congenic lymph nodes. IHC analysis revealed that the main producers of CCL11 in the lymph nodes were ED1^+^ and/or Iba-1^+^ macrophages, as well as plasma cells but none of the CCL11^+^ cells were Ox6^+^, CD11b^+^ or Ox62^+^ dendritic cells. We did not observe any CCL11 secreting CD3^+^ (CD4^+^ or CD8^+^) T lymphocytes or CD45RA^+^ B-cells (Figure 3B). CCR3 was expressed by W3/13^+^ lymphocytes, as well as by the ED1^+^ macrophage population. No CCR3^+^/CD45RA^+^ B-cells were detected in the peripheral lymph node tissue (Figure 3C).

### *Eae18b* Regulates the T Cell Response in EAE

**Figure 4:**
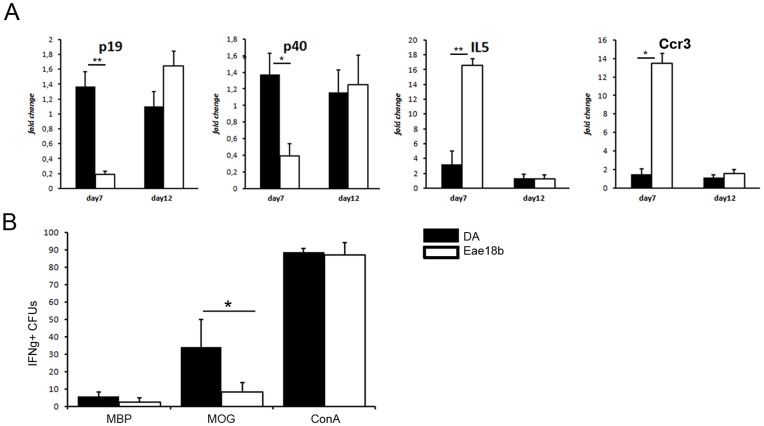
*Eae18b* modulates the immune response and reduces activation of MOG specific T cells. (A) Pro-inflammatory cytokines *p19*, *p40* were significantly upregulated in controls (DA), while congenic (Eae18b) animals upregulated anti-inflammatory *IL-5* cytokine as well as *CCR3*, main receptor for CCL11 chemokine (mRNA analysis of the inguinal lymph node tissue collected day 7 and 12 p.i.). Five to eight animals per strain were used in all time points analyzed and experiments were repeated twice. Error bars represent SEM, statistics were calculated using Mann-Whitney non-parametric test, (*) indicates p-value <0.05, (**) indicates p-value <0.01. (B) Elispot analysis revealed less pathogenic IFN-γ producing MOG specific T cells in the *Eae18b* strain; rat splenocytes harvested on day 15 and 21 p.i., representative data shown.

We further analyzed the mRNA expression levels of cytokines and receptors characteristic for either Th1/Th17 or Th2 response. *IL-5,* one of the key cytokines of the Th2 response, was upregulated in the lymph nodes of the congenic animals compared to DA controls (Figure 4A). In contrast, cytokines associated with a Th1/Th17 response, i.e. the IL-23 subunits *p19* and *p40,* were upregulated in the DA strain compared to the congenic strain (Figure 4A). We also observed a tendency for higher expression of *Gata-3* and *IL-4* in the lymph nodes of congenic animals, but the difference was not significant (data not shown). Additionally, the peripheral lymph node tissue from the congenic animals harvested on day 7 also showed significant upregulation of *Ccr3* mRNA (Figure 4A), the gene coding for the major CCL11 receptor [15]. Using flow cytometry, we analyzed the activation and recruitment of cells in the lymph nodes during the induction phase of the disease (day 7 p.i.), but could not detect any differences in major T cell subsets, B cells, macrophages or dendritic cells (Figures S2 and S3). Further, *in vitro* proliferative capability of lymphocytes (Figure S4), as well as specific anti-MOG antibody production (IgG1, IgG2b, IgG2c and total IgG, Figure S5) was also similar between the strains, indicating that the disease regulation by the congenic region is not primarily through regulation of these phenotypes. However, all those analysis have been performed during initial, or the early disease phase. In order to investigate if the specific response to MOG was altered in congenics in a later disease time-point, we performed an IFN-γ Elispot analysis on *ex vivo* splenocytes on day 15 and 21 p.i. The specific T cell response towards MOG antigen was 3-fold downregulated in the congenic rats compared to the controls (Figure 4B), whereas the response to the positive control Concavalin A and myelin basic protein (MBP), used as an unspecific antigen, was similar in both groups. These results indicate that *Eae18b* locus specifically modifies the T cell response by a Th2-like activation and suppression of the T cell expansion in response to MOG, thereby reducing the autoimmune response.

### *Eae18b* Reduces Cell Recruitment to the Inflammation Site

**Figure 5:**
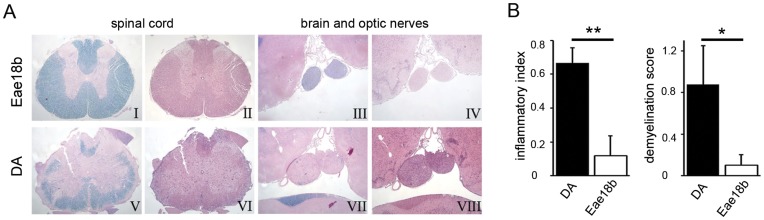
Congenic DA.PVG-*Eae18b* rats developed less severe neuroinflammation and demyelination. (A) Histopathological analysis of the group representative paraffin embedded tissue cross-sections from congenic and control spinal cord (I, II and V, VI, respectively) or brain and optic nerves (III, IV and VII, VIII, respectively) on day 15 p.i. (five congenic and four control animals analyzed). Sections were stained with Klüver (I, III, V, VII) to reveal demyelination and Hämalaun & eosin (II, IV, VI, VIII) to detect inflammatory infiltrates. The congenic strain showed minor subpial demyelination and cell infiltration in the spinal cord (I, II), while the brain and optic nerves remained intact (III, IV). In contrast, the control strain displayed confluent subpial demyelinated lesion of the spinal cord white matter (V, VI) as well as an extensive myelin loss in both optic nerves (VII) accompanied by recruitment of inflammatory cells (VIII). (B) Analysis of the material shown in A. Inflammatory index (I.I., first graph from left) and demyelination score (DM, second graph) are both significantly higher in DA controls compared to *Eae18b* congenic rats. Error bars represent SEM, and (*) indicates p-value <0.05, (**) indicates p-value <0.01 calculated by Students t-test.

**Figure 6:**
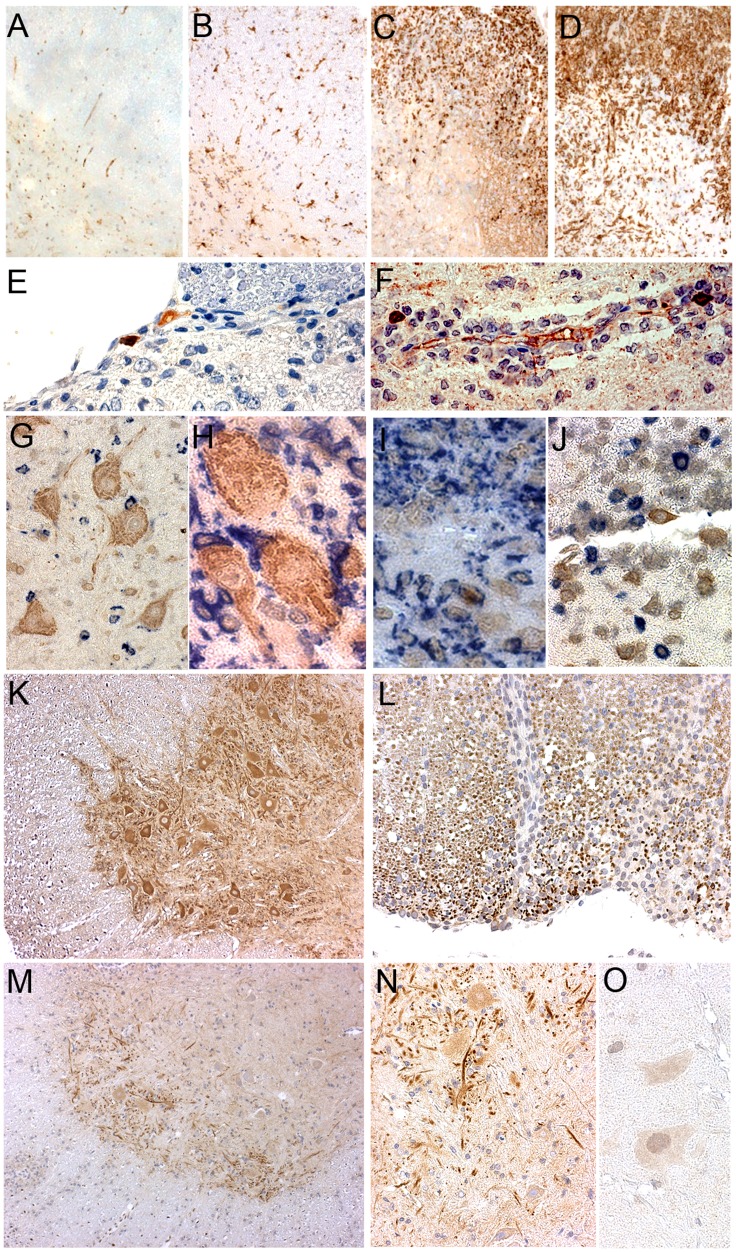
CCL11 and CCR3 expression in the central nervous system of *Eae18b* congenic rats. (A–D) Congenic and control group representative spinal cord cross sections stained for ED1 (A, C) and Iba-1 (B, D), both hallmarks for activation of microglia/macrophages (brown). Control rats (C, D) showed upregulation of both markers in contrast to congenics (A, B). (E, F) Portions of the control spinal cord cross section showing IgG^+^ single positive (golden brown, E) plasma cell and CCL11^+^/IgG^+^ double positive (maroon; E, F) plasma cells. (G, H) Congenic spinal cord; ventral horn gray matter portion showing CCL11 in neuronal pericarya, dendrites and axons (brown) together with co-stained ED1^+^ (G) or Iba-1^+^ (H) cells (both in blue). (I) Congenic spinal cord portion presenting solitary CCL11^+^ (golden brown)/ED1^+^ (blue) macrophage on the inflammation site. (J) CCL11^+^ plasma cell (golden brown) not co-localizing with CD3^+^ T cells (blue), here in the control spinal cord cross section. (K) Ventral horn of the congenic spinal cord gray matter not undergoing inflammation showing CCR3 upregulation in neurons, dendrites and axons (brown). (L) Dorsal spinal cord white matter expressing CCR3 (dark brown dots), partially downregulated within demyelinated lesion on the right side of the image. (M, N) Ventral horn of the congenic spinal cord gray matter undergoing inflammation revealing CCR3 downregulation in neurons (brown, in the lower (M, 40×) and a chosen detail in the higher magnification (N, 160×). (O) Negative control; a detail representing resident cells in the ventral horn of the spinal cord gray matter.

To assess the consequences of the lower disease score in the congenic animals undergoing EAE, we performed histopathological analysis of the brain and spinal cord on day 12 and 15 p.i. (Figure 5A; histopathological analysis for day 12 not shown). No congenic animal showed signs of inflammation/demyelination in the brain, although spinal cord tissue was affected. In contrast to the congenic group, 75% of the control animals developed brain lesions predominantly in medulla, pons and in optic nerves (Figure 5A). Congenic CNS showed overall significantly reduced inflammation (here referred to as inflammatory index I.I., according to [5]) and demyelination (presented as demyelination score, DM) compared to the controls (Figure 5B). Moreover, IHC analysis of the spinal cord cross-sections in both time-points showed that the control group had cumulatively more ED1^+^ and Iba-1^+^ macrophages/brain resident activated microglia, which correlated with their generally higher disease severity (Figure 6A–D). On day 15 p.i. one congenic animal with similar disease severity/neuroinflammation parameters as the controls, recruited surprisingly low amount of ED1^+^ peripheral macrophages to the demyelinated lesions, while the number of Iba-1^+^ activated brain resident microglia and macrophages was comparable to those observed in controls (data not shown).

In both strains we observed CCL11^+^ neurons located in the ventral horns of the spinal cord gray matter. CCL11 protein was detected in neuronal pericarya, dendrites and axons (Figure 6G and H). Furthermore, rare CCL11^+^ plasma cells were also detected in the inflamed spinal cord tissue (Figure 6E and F). No CCL11^+^ plasma cells were detected in the congenics on day 12 p.i. while control spinal cord already started harboring these cells. On day 15 p.i. we could also find CCL11^+^ plasma cells in the congenic animals. However, they were very rare and significantly less numerous compared to controls (1±0,45 in the congenic vs. 14,75±3,17 in the controls; p = 0,0009; MV±SEM, number of cells per spinal cord cross section). We also observed several CCL11-producing ED1^+^ macrophages recruited to inflammatory lesions, predominantly in the congenic group (Figure 6I), while no CCL11 co-localization was observed with CD3^+^ T cells (Figure 6J).

It has been described that eosinophils are the main responders to CCL11 at inflammatory sites in asthma [16], but also in neuromyelitis optica [17]. In order to investigate if the increased CCL11 expression in the congenic animals also correlated with eosinophils recruitment in our animal model, we stained the spinal cord cross sections with an antibody directed against eosinophil peroxidase (EPX). For that purpose we analyzed spinal cord cross-sections from both animal groups harvested on day 7–8, 12 and 15 p.i. The earlier time points were included in the analysis since eosinophils enter the CNS in a relatively short time-window, prior to massive invasion of the other inflammatory cells. However, we could not detect any eosinophils at any time-point analyzed (data not shown). Absence of eosinophils was also confirmed in sections stained for Hematoxylin & eosin. More interestingly, we observed significantly higher numbers of neutrophile granulocytes invading demyelinated lesions in the control animals (0,25±0,25 in the congenic vs. 27±11,02; p = 0,0168; MV±SEM, number of cells in whole spinal cord). Thus, in our congenic strain CCL11 seems to be involved in a distinct signaling pathway not related to the well-described eosinophil recruitment.

The presence of CCL11’s main receptor CCR3 in the CNS was observed in the ventral horns of the spinal cord gray matter as well as in the subpial white matter portion. CCR3 staining was restricted to neurons, dendrites and axons as well as to ependymal glia cells and was particularly intense in the ventral horn neurons of the congenic animals not undergoing neuroinflammation (Figure 6K). However, affected spinal cord tissue from both strains tended to downregulate CCR3^+^ expression in response to inflammation (Figure 6L–N). Additionally, we could not detect co-localization of CCR3 with any other brain resident or infiltrating cells (data not shown). Overall, in our MS-model congenic animals developed milder disease, associated with a reduced recruitment of ED1^+^and W3/13^+^ cells. The lower clinical score was reflected in a smaller size of less numerous demyelinated lesions in the CNS of the protected congenic strain, which also harbored significantly less granulocytes and plasma cells compared to EAE-susceptible DA controls.

### *Eae18b* Influences BBB Homeostasis

**Figure 7:**
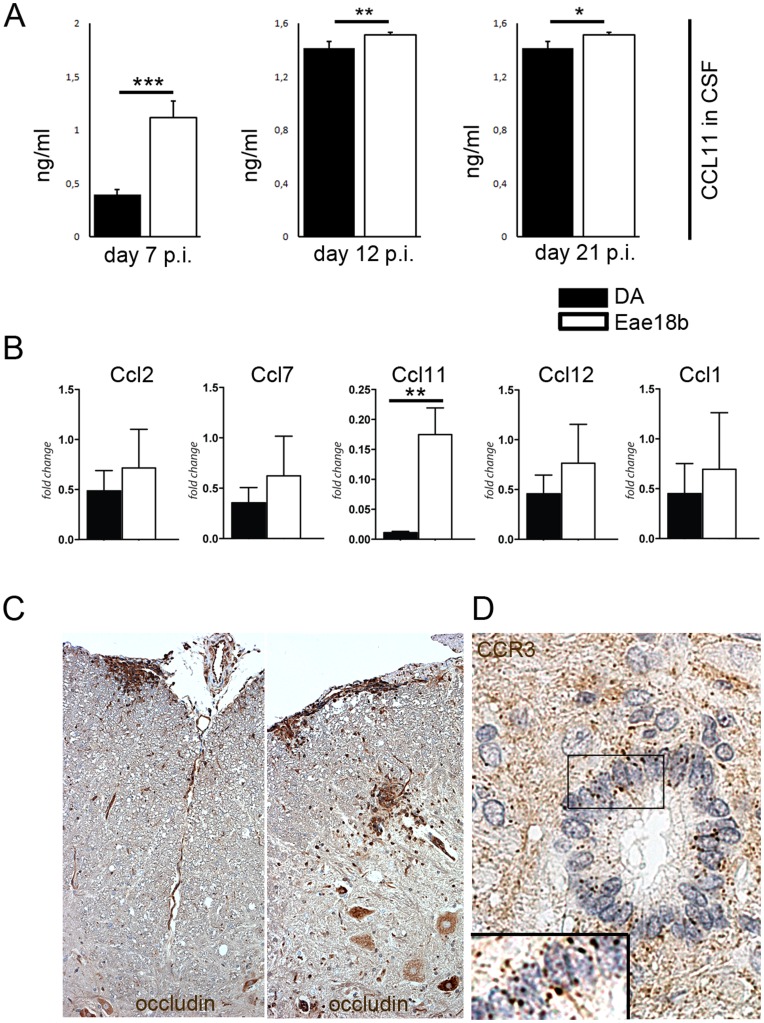
*Eae18b* regulates intrathecal production of CCL11 and influences the BBB integrity. (A) During the whole disease course CCL11 protein levels were increased in the cerebrospinal fluid (CSF), in the congenics (Eae18b) compared to controls (DA). Experiments repeated twice, three to five animals per group analyzed. (B) mRNA levels of *Ccl11* measured in the late disease time point (day 23 p.i.) showed upregulation in the congenic spinal cord. Error bars represent SEM, and (*) indicates p-value <0.05, (**) indicates p-value <0.01, (***) indicates p-value <0.001 calculated by Student’s t-test in (A) and by Mann-Whitney non-parametrical test in (B). (C) Representative images of occludin, a marker for the BBB tight junction components; more abundant in the congenic spinal cord (cross sections of seven congenic and eight control animals analyzed on day 12 p.i.). (D) Representative image of CCR3, the main receptor for CCL11, expressed on ependymal cells of the spinal cord canalis centralis, including a detail in high magnification (160×). Five congenic and four control animals analyzed on day 15 p.i.

In order to identify possible mechanism how elevated CCL11 levels in the CNS protect against EAE, we analyzed CCL11 concentration in serum and CSF on days 7, 12 and 21 p.i. by enzyme-linked immunosorbent assay (ELISA). We detected significantly higher levels of CCL11 in congenic CSF in all analysed time points (Figure 7A) while CCL11 levels in blood serum were comparable in both groups through the whole disease course (Figure S6). Higher amounts of CCL11 in the congenic CSF correlated with increased mRNA expression in spinal cord on day 7, 12 (Figure 2B) and 23 p.i. (Figure 7B). This suggests a central protective effect of the congenic fragment through an enhancement of intrathecal chemokine production. Additionally, we detected the main CCL11 receptor, CCR3, expressed on CSF- producing ependymal cells of the spinal cord’s canalis centralis (Figure 7D).

We suspected that differential levels of CCL11 measured in the CSF might have an impact on the BBB integrity. As a read-out for determination of the BBB property, we used dysferlin, a sensitive marker for “leaky” brain blood vessels [18], and occludin, a marker frequently used for detecting one of the main tight junction components [19]. IHC analysis of the CNS performed on day 12 p.i., i.e. prior to the infiltration of inflammatory cells from the periphery to the inflammatory lesions, revealed more occludin^+^ blood vessels in the congenic animals (12±5,26 in the congenic vs. 3±1,25 in the controls; p = 0,04; MW± SEM, number of cells per spinal cord cross section; Figure 7C), while the blood vessels in controls tended to express higher levels of dysferlin (data not shown). Occludin upregulation was also observed in perivascular and subpial regions related to inflammatory lesions. Our observations suggest that the *Eae18b* locus regulates intrathecal production of CCL11, and that the increased chemokine levels in the congenic animals are associated with maintenance of the integrity of the blood-brain barrier.

## Discussion

Previous linkage analyses identified the *Eae18b* QTL on rat chromosome 10, which regulates severity of MOG-EAE [9], [12]. The region includes a cluster of chemokine genes that are also associated with susceptibility to MS [12], [13], [20], [21], [22]. Although this region has not been confirmed in recent whole genome studies in MS [2], we believe that detailed studies of biological effects of naturally occurring genetic variants can identify important regulatory mechanisms in neuroinflammation. In this study we developed and tested a congenic strain carrying the resistant PVG alleles in the *Eae18b* region on the susceptible DA background, to gain further insights in the effects of genetically determined immune regulation by the chemokine genes in the cluster (*Ccl2, Ccl7, Ccl11, Ccl12* and *Ccl1*). The congenic strain exhibited milder disease with less severe inflammation and demyelination in the CNS after MOG immunization. Experiments on the congenic strain thereby confirmed previous statistical linkage analysis and provided a biological system to test immunological mechanisms regulated by the susceptible and resistant *Eae18b* alleles.

We have previously shown that the DA and PVG.av1 strains have identical sequence in the exons of the chemokine genes, indicating that genetically determined expression differences underlie the protection against EAE [13]. Therefore we focused first on the gene expression of chemokines present in the *Eae18b* congenic region. *Ccl11* expression, but no other chemokine genes in the cluster, was significantly upregulated in spinal cords and peripheral lymph nodes of the EAE-protected DA.PVG-*Eae18b* congenic strain compared to the susceptible DA control. To unravel in more detail how the defined genetic region mediates the protective effect, we performed extensive IHC analysis to determine which cells produce CCL11 in the CNS, and in the peripheral lymph nodes. In EAE, the autoimmune activation is initiated in the draining inguinal lymph nodes after immunization with MOG. Lymph node tissue analysis prior to clinical disease onset (day 7 p.i.) showed upregulation of CCL11 chemokine protein in the congenic strain. Co-staining for CCL11 with markers for various immune cell types showed that CCL11 is mainly produced by ED1^+^ and Iba1^+^ macrophages in the local draining lymph nodes whereas T cells (CD4^+^ and CD8^+^), and CD45RA^+^ B cells did not produce CCL11. Additionally, we could not detect CCL11 production in any of lymph node resident CD11b^+^ or Ox62^+^ dendritic cells. The absence of MHC class II expression on the surface of the CCL11-producing macrophages suggests that in our MS model, these cells are involved in modulation of the local microenvironment, rather than specific antigen presentation.

CCL11 and its main receptor CCR3 are effectors of the Th2 response [15] and CCR3 has been shown to be expressed on Th2 cells, but also on eosinophils, dendritic cells, basophils and neurons [15], [23], [24], [25], [26], [27]. We observed upregulated *Ccr3* mRNA and protein in the congenic lymph nodes, expressed on ED1^+^ macrophages and W3/13^+^ T lymphocytes. Previous studies have shown that CCL11 can inhibit dendritic cell maturation *in vitro* and reduce the endocytotic capacity of these cells, through induction of SOCS1 and 3 [28]. Even though the effects of CCL11 on monocyte-derived cell populations in EAE needs to be investigated further, similar mechanisms could be involved in the protection against disease that is seen in the congenic strain. In addition, our data suggest that CCL11 and CCR3 contribute to a preferential Th2 recruitment and/or activation in the congenic lymph tissue, exemplified by the upregulation of *IL-5* and downregulation of *IL-23* in the congenic strain. In contrast, the susceptible DA strain showed a typical Th1/Th17 inflammatory response, including high expression of both IL-23 subunits, previously implicated in EAE development [29], [30]. *In vitro* proliferation and anti-MOG IgG levels in serum were similar in the both strains, whereas we observed a 3-fold reduction of IFN-γ producing MOG specific T cells in the congenic strain. Taken together, the data indicate that both strains have the ability to respond to inflammatory stimuli but the response is qualitatively different, reflected by the differential expression of pro- and anti-inflammatory cytokines in the lymph nodes as well as by the reduced number of IFN-γ producing MOG specific T cells in the congenic animals.

Analysis of the CNS, the target organ for EAE revealed that the milder EAE phenotype in the congenic strain was accompanied by significantly lower myelin loss, reduced cumulative microglia activation, and a lower macrophage and lymphocyte recruitment to the brain and spinal cord of the congenic animals. CCL11 is traditionally associated with eosinophil recruitment and pro-inflammatory response, as seen in asthma [16] or associated with highly destructive CNS lesions observed in neuromyelitis optica [17]. In contrast to these observations, CCL11 has also been implicated as a regulator of monocyte activation and differentiation, apart from chemotaxis [28]. In our model, we did not observe any eosinophil invasion and related CNS tissue destruction, but instead we observed a significant increase in neutrophil granulocytes in inflammatory lesions of the control compared to the congenic animals. The upregulation of CCL11 in the CNS might instead have influenced the neuronal function and homeostasis, as these cells produced both CCL11 and CCR3. It has previously been shown that CCR3 might have a role in neuroprotection, Ccr3^−/−^ mice have significantly reduced survival after facial nerve axotomy, possibly through a Th2-related mechanism [31].

The levels of CCL11 measured in the CSF revealed significantly increased intrathecal production of this chemokine in the congenic strain during the whole disease course (day 7, 12 and 21 p.i.), while the levels in blood serum were comparable between the strains in all analyzed time-points. Higher amounts of CCL11 in the congenic CSF correlated with increased mRNA levels in spinal cord on day 7, 12 and 23 p.i. These data suggest that continued high CCL11 secretion through the whole disease course contributed to a milder disease profile observed in the congenic strain. In this context we also analyzed the influence on BBB integrity and could observe an increased expression of occludin in the lesions and lesion related blood vessels in the congenic strain, which indicates maintenance of BBB integrity and thus reduced invasion of inflammatory cells into the CNS.

In conclusion, we present a biological confirmation of the genetic regulation of EAE by the *Eae18b* locus on rat chromosome 10, and implicate differential regulation of *Ccl11* in CNS and periphery as a mechanism underlying the disease protection. We propose that the increased CCL11 expression and secretion by macrophages in the peripheral lymph nodes represent an early step in an active signaling pathway involving the CCR3 receptor, which leads to a preferential activation of the Th2 immune response. In addition, the congenic region also contributes to high levels of CCL11 in the CSF, maintained BBB integrity and reduced number of inflammatory cells in the CNS, indicating additional regulatory mechanisms in the target organ. Further studies are needed do define the details of the regulatory pathways involved in this diseases protection, but this study indicates that modulation of *Ccl11* expression and downstream signaling through CCR3 could provide efficient strategies for immuno-modulation in MS and other inflammatory diseases.

## Materials and Methods

### Ethics Statement

All experiments in this study were approved by the North Stockholm Animal Ethics Committee (Stockholms Norra djurförsöksetiska nämnd) (permit numbers: N332/06, N338/09, N15/10, N65/10) and performed in accordance with the guidelines from the Swedish National Board for Laboratory Animals and the European Community Council Directive (86/609/EEC). In accordance with the ethical permits, the animals were examined at least daily for clinical signs of EAE and signs of distress, dehydration or underfeeding. To ensure proper food intake in partially paralyzed animals, all cages were supplemented with wet chow throughout the experiments. In addition, animals were euthanized if they lost more than 25% body weight during the experiment, or remained tetraplegic (score 4, see below) or showed signs of severe balance disturbance for 2 consecutive days.

Rats were routinely tested according to a health-monitoring program at the National Veterinary Institute (Statens Veterinärmedicinska Anstalt, SVA) in Uppsala, Sweden. All animals were bred and housed at the Karolinska University Hospital (Stockholm, Sweden), in polystyrene cages containing aspen wood shavings and with free access to water and standard rodent chow, with a 12-hour light/dark cycle.

### Animals

Inbred DA rats were originally obtained from the Zentralinstitut für Versuchstierzucht (Hannover, Germany) and PVG.av1 rats from Harlan UK Limited (Blackthorn, UK). Animals for congenic breeding were selected from the 10^th^ generation of an advanced intercross line (AIL) [32] originating from the EAE-susceptible DA and EAE-resistant PVG.av1 rat strains. Selected males containing a PVG.av1 fragment in the *Eae18b* region (from OT24.18 to D10Rat93 marker, at 68.36 Mb and 81.9 Mb, respectively) were backcrossed with DA females for 8 generations, and offspring were genotyped using microsatellite markers in each generation. Genotyping of animals was performed on DNA extracted from ear tissues according to a standard protocol. PCR amplification was performed with VIC, NED, FAM or PET end-labeled forward primers (Applied Biosystems, Carlsbad, CA). The PCR products were size fractioned using an ABI 3730 capillary sequencer and analyzed in GeneMapper v3.7 (Applied Biosystems). In the 8^th^ generation, heterozygous males and females were crossed to obtain the experimental population of homozygous congenic rats and littermate controls.

### EAE Induction and Phenotypic Evaluation

Recombinant MOG (aa 1–125 from the N-terminus) was expressed in *Escherichia coli* and purified to homogeneity by chelate chromatography as previously described [33]. The purified protein, dissolved in 6 M urea, was dialyzed against PBS to obtain a semi-precipitated preparation that was stored at −20°C. Rats between 10 and 14 weeks of age were anesthetized with isofluorane (Forene, Abbott Laboratories, Abbot Park, IL) and immunized with a single subcutaneous injection in the dorsal base of the tail with 200 µl of inoculum containing rMOG (aa 1–125), 15 µg/rat (females) or 30 µg/rat (males), in saline emulsified with IFA (Sigma Aldrich, St. Louis, MO), ratio 1∶1. The rats were weighed and monitored daily for clinical signs of EAE. The clinical score was graded as follows: 0, no clinical signs of EAE; 1, tail weakness or tail paralysis; 2, hind leg paraparesis or hemiparesis; 3, hind leg paralysis or hemiparalysis; 4, tetraplegia or moribund; 5, death. The following clinical parameters were assessed for each animal: EAE incidence (clinical signs for >1 day), maximum EAE score (the highest clinical score observed during EAE), cumulative EAE score (the sum of daily clinical scores).

### Quantitative Real-time PCR

Animals were euthanized using CO_2_ at selected time-points. Draining inguinal lymph nodes and cervical spinal cords were collected and transferred on to dry ice and kept at −70°C until isolation of mRNA using RNeasy mini kit (Qiagen, Hilden, Germany), including on-column DNA-digestion. Subsequent reverse transcription was performed using or iScript cDNA Synthesis Kit (BioRad Laboratories, Hercules, CA). All primers used for quantitative real-time PCR in the study are found in Table S1. Quantitative real-time PCR was performed using a BioRad iQ5 iCycler Detection System (BioRad Laboratories) with a three-step PCR protocol (95°C for 10 min, 40 or 50 cycles of 95°C for 15 sec, 60°C for 30 sec. and 72°C for 30 sec, followed by ramping from 55°C to 95°C at 1°C/minute for melt curve analysis), using SYBR green as fluorophore (BioRad Laboratories). Relative expression levels, corrected for amplification efficiency, were recorded in iQ5 v2.0 software (BioRad Laboratories) and are presented as fold change compared to the mean expression of the target in the experiment, with *Gapdh* as reference gene, using the formula 2^−ΔΔCt^.

### Histopathological and IHC Analysis

On day 7, 8, 12 and 15 post immunization each 4–8 littermate controls and 4–8 DA.PVG-*Eae18b* animals were euthanized using CO_2_ and perfused via the left heart ventricle with PBS followed by 4% paraformaldehyde. Paraformaldehyde-fixed 3 to 5 mm thick paraffin embedded sections of the brain and spinal cord were dewaxed in xylol, rehydrated and then stained with Hematoxylin & eosin and Luxol fast blue to assess tissue inflammation and demyelination, respectively. The inflammatory index (I.I) and demyelination score (DM) were determined by the number and size of demyelinated lesions of each animal on an average of 12 complete brain and spinal cord cross-sections as previously described [5]. In adjacent serial sections IHC analysis was performed using antibodies against the following targets: α- CD68 (ED1, mouse anti-rat, AbD Serotec, 1∶1000), α-Iba1 (AIF1, mouse anti-rat, Millipore, 1∶1000), CD43 (W3/13 mouse anti-rat, Abcam, 1∶100), α-GFAP (astrocyte marker, mouse anti-rat, Abcam, 1∶200), α-CD3 (rabbit anti-human, cross-reactive to rat CD3; Dakopatts, Denmark), α-rat IgG (biotinylated, GE Healthcare, 1∶200), α-eotaxin (CCL11 (C-19) goat anti-rat, Santa Cruz BT, 1∶300), α-CCR3 (rabbit anti-rat, Abcam, 1∶300), α-Dysferlin (Ham1/7B6, mouse anti-rat, Abcam, prediluted), α-Occludin (rabbit anti-rat, Abcam 1∶100), α-CD4 (W3/25 Biotin, mouse anti-rat, Abcam, 1∶100), α-CD8α (Ox-8 mouse anti-rat, AbD Serotec, 1∶200), α-Ox6 (MHC class II, mouse anti-rat, AbD Serotec, 1∶150), α-CD11b (mouse anti-rat, BD Pharminogen, 1∶100), α-Ox62 (mouse anti-rat, AbD Serotec, 1∶100), α-CD45R (B220 mouse anti-rat, eBioscience, 1∶200) and EPX (H-75), Santa Cruz BT, 1∶100). Secondary antibodies used were biotinylated (Amersham Pharmacia Biotech, 1∶200) or alkaline phosphatase-conjugated (Dakopatts, Denmark, 1∶200).

For IHC, paraffin sections of the spinal cord and inguinal lymph nodes were treated as previously described [34], deparaffinized in xylol and transferred to 90% ethanol. Endogenous peroxidase was blocked by incubation in methanol with 0.02% H_2_O_2_ (30 minutes, room temperature). Sections were then transferred to distilled water via a 90%, 70%, and 50% ethanol series. Antigen retrieval was performed with ethylenediamine tetraacetic acid buffer, pH 8.5, or citrate buffer pH 6.0 by warming for 60 minutes in a household steamer device (Braun, Germany). For detection of IgG sections were treated with 0.03% protease from Streptomyces griseus (Sigma) for 15 minutes at 37°C. Sections were incubated with 10% fetal calf serum in 0.1 mol/L PBS prior to incubation with primary antibody on 4°C, overnight. For double staining (co-staining) we used primary antibodies from two different species. After washing in PBS, sections were incubated with a mixture of one alkaline phosphatase-conjugated and one biotinylated secondary antibody in 10% fetal calf serum in PBS for 1 hour at room temperature. Finally, avidin peroxidase (1∶100, Sigma, Vienna, Austria) was applied for 1 hour at room temperature. Alkaline phosphatase label was visualized with Fast Blue B base or Fast Red TR Salt (Sigma) and avidin peroxidase with 3,3′diaminobenzidine-tetrahydrochloride (DAB, Sigma). Simple stainings were performed with biotin-avidin peroxidase detection system and visualized with DAB. Control sections were incubated in the absence of primary antibodies. Evaluation has been performed on at least 12 whole spinal cord cross sections. Imaging was performed on a Leica Polyvar 2 microscope.

### Western Blot

Inguinal lymph nodes were harvested on day 7 p.i., frozen in liquid nitrogen and kept at −80°C. Tissue were homogenized in the lysis buffer (50 mM Tris-HCl pH 8.0, 150 mM NaCl_2_, 1% Triton-X100, 0,5% deoxycholic acid and 0.1% SDS) supplemented with Complete Protease Inhibitor Cocktail (Roche). Tissue lysates were incubated on ice for 30 min followed by centrifugation at 16.000 *g* at 4°C for 20 min where after the lysates were subjected to SDS–PAGE on NuPage 12% Bis-Tris gels (Invitrogen) under reducing conditions. Separated proteins were blotted onto filters using the iBlot system (Invitrogen). Membranes were blocked for 1 h in Tris-buffered saline containing 0.1% Tween-20 (TBST) and 5% bovine serum albumin (BSA) followed by overnight incubation at 4 C with goat polyclonal anti-CCL11 antibody (Santa Cruz BT, 1∶ 1000 in TBST, 1% BSA), rabbit monoclonal CCR3 (Abcam, 1∶ 1000 in TBST, 1% BSA) or mouse monoclonal b-actin (1∶ 1000 in TBST, 1% BSA). Membranes were incubated with horseradish peroxidase conjugated antimouse IgG (dilution 1∶2000; Amersham Pharmacia Biotech.) for 1 h at room temperature and visualized by enhanced chemiluminescence (ECL, Amersham Pharmacia Biotech.). The optical density of the respective bands was digitized and analysed using the Biorad Geldoc XR (Biorad).

### Elispot

Spleens were harvested on day 15 and 21 p.i. and placed in DMEM before being mechanically separated by passage through a mesh screen with the bolus of a syringe. Splenocytes from DA and DA.PVG-*Eea18b* (n = 4 for each condition) were used for IFN-γ Elispot analysis (R&D systems) in a 96 well plate. ConA (12.5 ng/well), MOG (50 ng/well) and MBP (50 ng/well) were used to stimulate 50.000 splenocytes/well in total volume of 100 µl. After 48 hours incubation IFN-γ producing cells were visualized by antibody staining according to the manufacturer’s protocol and the *colony* forming units (CFU) were visualized using an AID Elispot reader (AID, Germany).

### ELISA

Cerebrospinal fluid (CSF) was harvested on day 7, 12 and 21 p.i., frozen in liquid nitrogen and stored at −70°C until used for ELISA. Samples and standards were incubated together with CCL11 conjugate according to the manufacturer’s protocol (Rat eosinophil chemotactic factor kit, Emelcabio, Netherlands).

### Statistical Analysis

Mann-Whitney non-parametrical test was used for statistical analysis, if not stated otherwise. For all tests p-values <0.05 were considered significant. All statistical analyses were performed in GraphPad Prism versions 4 or 5 (GraphPad, San Diego, CA). Mean values with standard error are presented in graphs, if not stated otherwise.

### Supporting Information

Description of flow cytometry, proliferation assay, determination of anti-MOG isotypes and CCL11 measurement in serum are found in Materials and Methods S1.

## Supporting Information

Figure S1 **Differental expression of chemokines in (A) inguinal lymph nodes and (B) spinal cord of parental DA and PVG.av1 rats.** Expression of mRNA from naïve animals (4 animals/strain) and in tissues harvested day 5 (8 animals/strain), 7 (4 animals/strain) and 12 (8 animals/strain) post immunization. Error bars represent SEM, Mann-Whitney non-parametric test was used for statistical analysis. P-values <0.05 were considered significant and are indicated in the figure. nd: not detected.
(EPS)

Figure S2 **DA and DA.PVG-** ***Eae18b*** **strains do not differ in proportions of main cell types (A) or subsets of T-cells (B).** Cell from lymph nodes from 5 animals/strain were collected day 5, 7 and 12 post immunization and analyzed using flow cytometry. Error bars represent SEM, and Mann-Whitney non-parametric test was used for statistical analysis. No significant differences were found. n.a: not analyzed.
(EPS)

Figure S3 **DA and DA.PVG-** ***Eae18b*** **strains do not differ in proportions of immune cell types.** Analysis performed by flow cytometry on cells collected from the lymph nodes on day 7 (A) and 12 (B) after immunization, 5 to 8 animals in each group. Y-axis presents the percentage (%) of detected cells out of all gated viable cells; error bars show Standard Deviation and Mann-Whitney non-parametric test was used for statistical analysis. No significant differences were found.
(TIF)

Figure S4 ***In vitro*** **proliferation reveals no differences between DA and DA.PVG-** ***Eae18b*** **rat strains.** Proliferation assay performed on lymphocytes collected day 7 post immunization from 5 DA and 5 DA.PVG-*Eae18b* females. Cells were stimulated for 18 hours with MOG (19 µg/ml), ConA (0.5 µg/ml) or media alone. Error bars represent SEM, and Mann-Whitney non-parametric test was used for statistical analysis. No significant differences were found between strains.
(EPS)

Figure S5 **DA and DA.PVG-** ***Eae18b*** **strains do not differ in secretion of specific antibodies.** Anti-MOG specific antibody production of IgG1, IgG2b, IgG2c and total IgG isotypes measured using ELISA in sera collected day 12 post immunization from DA (12 females and 9 males), and DA.PVG-*Eae18b* (11 females and 8 males). Error bars represent SEM, and Mann-Whitney non-parametric test was used for statistical analysis. No significant differences were found.
(EPS)

Figure S6 **DA and DA.PVG-** ***Eae18b*** **(Eae18b) rats have similar amounts of CCL11 in serum, at different time points after MOG immunization.** Experiments repeated twice, 3–5 animals per group analyzed. Error bars represent SEM, Students t-test was used for statistical analysis. No significant differences were found.
(TIF)

Table S1 **Sequences of primers used for quantitative real-time PCR, designed using Primer Express software.** The second pair of *Ccl11* [Ccl11 (2)] primers was used for confirmation, excluding potential artifacts originating from annealing differences in the two strains.
(DOC)

Materials and Methods S1 **Description of methods related to the supporting figures, including cytometry, proliferation assay, determination of anti-MOG isotypes and CCL11 measurement in serum.**
(DOC)
