# Supplementary figures and images for: Expression of Ccl11 Associates with Immune Response Modulation and Protection against Neuroinflammation in Rats
Source: PLoS One. 2012 Jul 16;7(7):e39794. doi: 10.1371/journal.pone.0039794 (PMC3397980; doi:10.1371/journal.pone.0039794)

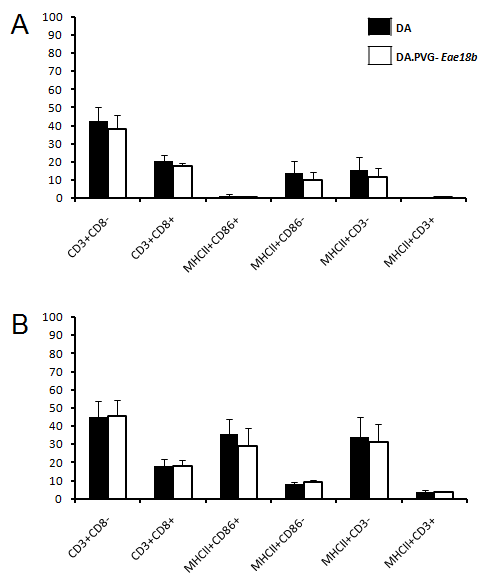

Supplement: Figure S3 — DA and DA.PVG- Eae18b strains do not differ in proportions of immune cell types. Analysis performed by flow cytometry on cells collected from the lymph nodes on day 7 (A) and 12 (B) after immunization, 5 to 8 animals in each group. Y-axis presents the percentage (%) of detected cells out of all gated viable cells; error bars show Standard Deviation and Mann-Whitney non-parametric test was used for statistical analysis. No significant differences were found. (TIF) [file pone.0039794.s003.tif]

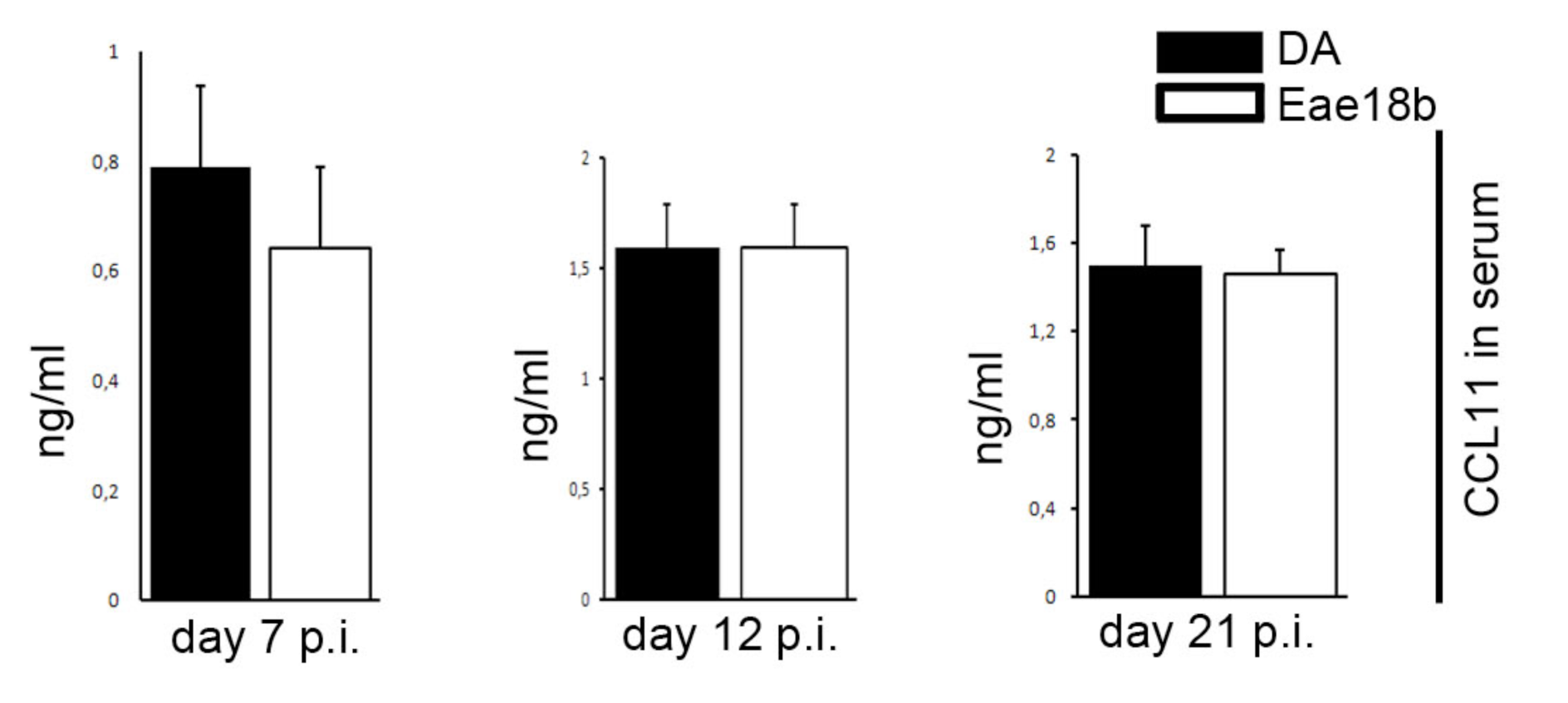

Supplement: Figure S6 — DA and DA.PVG- Eae18b (Eae18b) rats have similar amounts of CCL11 in serum, at different time points after MOG immunization. Experiments repeated twice, 3–5 animals per group analyzed. Error bars represent SEM, Students t-test was used for statistical analysis. No significant differences were found. (TIF) [file pone.0039794.s006.tif]
