# Supplementary material for: Expression of Ccl11 Associates with Immune Response Modulation and Protection against Neuroinflammation in Rats
Source: PLoS One. 2012 Jul 16;7(7):e39794. doi: 10.1371/journal.pone.0039794 (PMC3397980; doi:10.1371/journal.pone.0039794)
Supplement: Table S1 — Sequences of primers used for quantitative real-time PCR, designed using Primer Express software. The second pair of Ccl11 [Ccl11 (2)] primers was used for confirmation, excluding potential artifacts originating from annealing differences in the two strains. (DOC) [file pone.0039794.s007.doc]

| **Gene** | **Forward primer** | **Reverse primer** |
| --- | --- | --- |
| *Ccl1* | GTGGACAGCAAGAGCATGCA | GCAATCTTGTTCTCCAAGGTGTTC |
| *Ccl2* | TCTTGAGCTTGGTGACAAATACTACA | CCAATGAGTCGGATGGAGAA |
| *Ccl7* | GGCCTCCTCAACCCACTTCT | CCCTGGGAAGCTGTTATCTTCA |
| *Ccl11* | GCCATAGTCTTCAAGACCAAGCTT | TGGCATCCTGGACCCACTT |
| *Ccl11 (2)* | TCACCCTGATTGACCTGCAAC | ATTCTGGCTTGGCATGATGG |
| *Ccl12* | ccgggaagctgtgatcttca | Gacttctgatccaagtggttcatg |
| *IL-5* | TTCTGGCACCTCGGTTATGAA | AACAAGCCAGGTGCAACGA |
| *Ccr3* | TATCTTCCATGAGTCCCAAGAC | TTCTTAGAGCATGGAAACGC |
| *p19* | CCATGGAGAGCTTCATCTGTGT | TCGCTGCTGCCTTCCTGTA |
| *p40* | TCATCATGGACATCATCAAACC | GTGCTCCAGGAGTCAGGGTACT |
| *Β-actin* | CGTGAAAAGATGACCCAGATCA | AGAGGCATACAGGGACAACACA |
| *Gapdh* | TCAACTACATGGTCTACATGTTCCAG | TCCCATTCTCAGCCTTGACTG |
