# Supplementary material for: Expression of Ccl11 Associates with Immune Response Modulation and Protection against Neuroinflammation in Rats
Source: PLoS One. 2012 Jul 16;7(7):e39794. doi: 10.1371/journal.pone.0039794 (PMC3397980; doi:10.1371/journal.pone.0039794)
Supplement: Materials and Methods S1 — Description of methods related to the supporting figures, including cytometry, proliferation assay, determination of anti-MOG isotypes and CCL11 measurement in serum. (DOC) [file pone.0039794.s008.doc]

Materials and Methods S1

### Flow Cytometry

Lymph node cells were isolated as described above and washed with cold PBS and re-suspended in 200μl of PBS. Cells were stained for 20 min at 4^o^C with a combination of the following antibodies: CD3-APC, CD4-PE-Cy5, CD25-PE, MHCII-PercpCy5, CD86-FITC, CD25-PE, CD45ra-FITC and CD11b-FITC (BD Biosciences). Staining was visualized on a FACSort (BD, Franklin Lakes, USA) with Cell Quest (version 3.2.1f1, BD) and analyzed using FloJo (version 8.8).

### Proliferation assay

Lymph nodes were placed in DMEM enriched with 5% fetal calf serum, 1 % L-glutamine, 1% penicillin-streptomycin, 1% pyruvic acid and 50μM 2-Mercaptoethanol (complete media) before being mechanically separated by passing through a mesh screen with the bolus of a syringe. Cells were spun at 300g and re-suspended in complete media for counting. Cells were allocated to flow cytometry or cell culture. To measure proliferation 150,000 cells/well were plated in 96 well round bottom plates in triplicates. Cells were stimulated with either: complete media, 0.5 µg/ml Concanavalin A (Sigma-Aldrich) or 19 µg/ml MOG for 72 hrs at 37°C and 5% CO_2_. 18 hours before collection 1mCi of H^3^ radioactive thymidine (GE Healthcare, Bucks, UK) was added to each well. Cells were harvested using a Wallac Tomtec harvester (Perkin Elmer, Waltham, USA) and isotope incorporation measured using a Wallac TriLux 1450 MicroBeta (Perkin Elmer). Triplicates were averaged and a proliferation index calculated by dividing the stimulated average by the un-stimulated average for each individual animal.

### Anti-MOG isotype determination

Blood was collected day 12 post immunization from the tail vein under anaesthesia, and sera separated by centrifugation at +4°C. Anti-MOG antibody levels in sera were measured by enzyme-linked immunosorbent assay (ELISA). Ninety-six-well plates (Nunc) were coated with 10µg/ml of rMOG (100 µl/ well) overnight at 4 °C. Plates were washed with PBS/0.05% Tween20 and free binding sites were blocked with 5% fat-free milk in PBS/0.05% Tween20 for 1 h at room temperature (RT). After washing, diluted serum samples were added and incubated for 1 h at RT. Rabbit anti-rat total IgG (1:2000), IgG1 (1:1000), IgG2b (1:2000) or IgG2c (1:1000) (Nordic, Tilburg, Netherlands) were added for 1 h at RT after washing. Unbound antibodies were removed by washing prior to addition of a peroxidase-conjugated goat anti-rabbit antiserum (Nordic) (1:10000). After 30 min incubation, plates were washed and bound antibodies were visualized with 3,3′5,5′-tetramethylbenzidine (Sigma-Aldrich). The reaction was stopped by addition of 1 M HCl after 15 min incubation in darkness and the optical density was read at 450 nm. Serum samples from rats with high levels of each isotype detected in a pilot study were included on each plate to create a relative standard curve using seven 2-fold dilutions; the highest point was assigned 100 on the arbitrary unit scale.

**CCL11 determination in serum**Blood was collected and sera separated as above, on day 7, 12 or 21 p.i and frozen in liquid nitrogen and stored at -70 C until usage for ELISA. Samples and standards were incubated together with CCL11 conjugate according to the manufacturer’s protocol (Rat eosinophil chemotactic factor kit, Emelcabio, Netherlands).
